# Supplementary material for: Multicomponent Training Improves the Quality of Life of Older Adults at Risk of Frailty
Source: Healthcare (Basel). 2023 Oct 28;11(21):2844. doi: 10.3390/healthcare11212844 (PMC10650749; doi:10.3390/healthcare11212844)
Supplement: Supplementary file 1 [file healthcare-11-02844-s001.zip › healthcare-2661354-supplementary.pdf]

Supplementary material Table S1. Relationships between baseline body composition, physical fitness, nutritional variables values and total score in the EQ-5D-3L

	EQ-5D-3L overall index				EQ-VAS			
	r^2	Change r^2	Standardized β	p-value	r^2	Change r^2	Standardized β	p-value
Body composition measurements								
Weight(kg)	0.114	0.093	−0.372	**0.002**	0.019	0.001	−0.032	0.800
BMI (kg/cm^2)	0.185	0.168	−0.453	**<0.001**	0.032	0.013	−0.128	0.299
FM (kg)	0.223	0.174	−0.462	**<0.001**	0.040	0.003	−0.06	0.599
FFM (Kg)	0.019	0.003	−0.093	0.577	0.041	0.003	0.092	0.558
FM%	0.230	0.181	−0.514	**<0.001**	0.044	0.007	−0.101	0.431
Waist circum. (cm)	0.151	0.128	−0.421	**0.005**	0.045	0.021	−0.171	0.191
Hip circum. (cm)	0.120	0.119	−0.365	**0.004**	0.029	0.014	−0.125	0.329
Physical fitness variables								
Balance (s)	0.112	0.091	0.311	**0.002**	0.043	0.022	0.152	0.135
Arm Flexibility (cm)	0.076	0.055	0.241	**0.016**	0.042	0.021	0.148	0.143
Leg Flexibility(cm)	0.051	0.030	0.182	0.153	0.052	0.032	0.186	0.144
Leg Strength (rep)	0.148	0.127	0.363	**<0.001**	0.054	0.033	0.185	0.065
Arm Strength (rep)	0.074	0.050	0.237	**0.022**	0.048	0.027	0.172	0.100
Agility (s)	0.237	0.219	−0.499	**<0.001**	0.070	0.050	−0.231	**0.025**
Walking speed (s)	0.306	0.285	−0.550	**<0.001**	0.092	0.071	−0.276	**0.006**
Aerobic capacity (m)	0.269	0.254	0.532	**<0.001**	0.082	0.062	0.263	**0.012**
Handgrip Strength	0.089	0.049	0.339	**0.024**	0.054	0.015	0.209	0.209
Nutritional Variables								
MNA	0.135	0.072	0.274	**0.004**	0.051	0.008	−0.183	0.398
ADM	0.068	0.037	0.006	0.397	0.08	0.041	0.204	**0.048**

CON: control group, TRAIN: training group, EQ-5D-3L: EuroQol 5 Dimensions 3 Levels, BMI: Body Mass Index, FM: Fat Mass, FFM: Fat Free Mass, FM%: Fat Mass Percentage, Circum: Circumference, MNA: Mini Nutritional Assessment, ADM: Adherence to Mediterranean Diet, rep: repetitions. Significant p-values were set as <0.05.

Supplementary material Table S2a. Relationships between changes with training in body composition, physical fitness and changes in total score in the EQ-5D-3L

	CON				TRAIN			
	r^2	Change r^2	Standardized β	p-value	r^2	Change r^2	Standardized β	p-value
Body composition measurements								
Weight (kg)	0.072	0.034	−0.193	0.388	0.041	0.029	−0.177	0.269
BMI	0.080	0.041	−0.207	0.409	0.052	0.047	−0.221	0.240
FM (kg)	0.039	0.000	−0.004	0.988	0.062	0.050	−0.229	0.182
FFM (Kg)	0.099	0.060	−0.259	0.274	0.013	0.001	−0.030	0.867
FM%	0.054	0.015	0.135	0.585	0.032	0.020	−0.144	0.406
Waist circum. (cm)	0.024	0.001	0.029	0.926	0.077	0.072	−0.354	0.158
Hip circum. (cm)	0.217	0.060	−0.277	0.376	0.089	0.007	−0.087	0.644
Physical fitness variables								
Balance (s)	0.057	0.027	0.173	0.396	0.387	0.083	−0.296	0.058
Arm Flexibility (cm)	0.025	0.000	−0.019	0.922	0.067	0.000	−0.004	0.980
Leg Flexibility (cm)	0.080	0.056	0.237	0.229	0.086	0.009	−0.097	0.554
Leg Strength (rep)	0.033	0.003	0.057	0.776	0.071	0.002	−0.052	0.763
Arm Strength (rep)	0.049	0.013	−0.120	0.558	0.232	0.084	0.321	**0.048**
Agility (s)	0.070	0.039	0.204	0.323	0.069	0.029	−0.181	0.269
Walking speed (s)	0.123	0.093	0.305	0.110	0.071	0.070	−0.269	0.082
Aerobic capacity (m)	0.046	0.002	0.043	0.842	0.205	0.128	0.360	**0.018**
Handgrip Strength (kg)	0.048	0.018	0.136	0.493	0.257	0.190	−0.480	0.189

CON: control group, TRAIN: training group, EQ-5D-3L: EuroQol 5 Dimensions 3 Levels, BMI: Body Mass Index, FM: Fat Mass, FFM: Fat Free Mass, FM%: Fat Mass Percentage, Circum: Circumference, rep: repetitions. Significant p-values were set as <0.05.

Supplementary material Table S2b. Relationships between changes with training in body composition, physical fitness and changes in total score in the EQ-VAS

	CON				TRAIN			
	r^2	Change r^2	Standardized β	p-value	r^2	Change r^2	Standardized β	p-value
Body composition measurements								
Weight (kg)	0.242	0.001	0.040	0.838	.113	.101	−0.329	**0.048**
BMI	0.113	0.000	−0.012	0.961	0.122	0.083	−0.293	0.110
FM (kg)	0.247	0.000	−0.001	0.998	0.047	0.009	−0.098	0.568
FFM (Kg)	0.250	0.003	0.061	0.768	0.077	0.039	−0.209	0.232
FM%	0.080	0.078	0.302	0.209	0.116	0.006	−0.078	0.635
Waist circum. (cm)	0.139	0.010	−0.115	0.685	0.054	0.002	−0.057	0.818
Hip circum. (cm)	0.167	0.020	−0.157	0.605	0.101	0.051	−0.228	0.227
Physical fitness variables								
Balance (s)	0.138	0.008	0.498	0.623	0.016	0.000	−0.012	0.940
Arm Flexibility (cm)	0.145	0.001	0.023	0.902	0.056	0.040	−0.221	0.207
Leg Flexibility (cm)	0.173	0.028	−0.169	0.363	0.043	0.022	0.157	0.352
Leg Strength (rep)	0.132	0.002	−0.044	0.818	0.041	0.025	−0.174	0.328
Arm Strength (rep)	0.111	0.048	−0.228	0.255	0.033	0.021	−0.160	0.371
Agility (s)	0.150	0.044	0.071	0.716	0.040	0.023	0.162	0.336
Walking speed (s)	0.133	0.003	−0.055	0.766	0.017	0.001	0.034	0.832
Aerobic capacity (m)	0.147	0.005	−0.071	0.728	0.034	0.013	−0.117	0.471
Handgrip Strength (kg)	0.112	0.000	−0.010	0.960	0.021	0.005	0.430	0.670

CON: control group, TRAIN: training group, EQ-5D-3L: EuroQol 5 Dimensions 3 Levels, BMI: Body Mass Index, FM: Fat Mass, FFM: Fat Free Mass, FM%: Fat Mass Percentage, Circum: Circumference, rep: repetitions. Significant p-values were set as <0.05.
